# Role of Purified Anthocyanins in Improving Cardiometabolic Risk Factors in Chinese Men and Women with Prediabetes or Early Untreated Diabetes—A Randomized Controlled Trial

**DOI:** 10.3390/nu9101104

**Published:** 2017-10-10

**Authors:** Liping Yang, Wenhua Ling, Yan Yang, Yuming Chen, Zezhong Tian, Zhicheng Du, Jianying Chen, Yuanling Xie, Zhaomin Liu, Lili Yang

**Affiliations:** 1 Guangdong Provincial Key Laboratory of Food, Nutrition and Health, Department of Nutrition, School of Public Health, Sun Yat-sen University, Guangzhou 510080, China; yanglp6@mail2.sysu.edu.cn (L.Y.); lingwh@mail.sysu.edu.cn (W.L.); yangyan3@mail.sysu.edu.cn (Y.Y.); shishujushi@sina.com (Z.T.); 2 Department of Medical Statistics & Epidemiology, School of Public Health, Sun Yet-Sen University, Guangzhou 510080, China; chenyum@mail.sysu.edu.cn (Y.C.); duzhch3@mail2.sysu.edu.cn (Z.D.); 3 BaiYun Hospital, YueXiu District, Guangzhou 510030, China; jianyc@163.com (J.C.); xinling_3@aliyun.com (Y.X.)

**Keywords:** anthocyanin, cardiometabolic risk factors, oral glucose tolerance test, prediabetes

## Abstract

**Objective:** In vitro and animal studies suggest that purified anthocyanins have favorable effects on metabolic profiles, but clinical trials have reported inconsistent findings. Furthermore, no study has been specifically conducted among individuals with prediabetes. The aim of this study was to investigate whether purified anthocyanins could improve cardiometabolic risk factors in Chinese adults with early untreated hyperglycemia. **Research Design and Methods:** This was a 12-week randomized, double-blind, placebo-controlled trial. A total of 160 participants aged 40–75 years with prediabetes or early untreated diabetes were randomly allocated to receive either purified anthocyanins (320 mg/day, *n* = 80) or placebo (*n* = 80) of identical appearance. A three-hour oral glucose tolerance test (OGTT) was performed, and cardiometabolic biomarkers (glycated hemoglobin A1c (HbA1c), fasting and postprandial glucose, insulin, C-peptide, and lipids) were measured at baseline and at the end of the trial. **Results:** A total of 138 subjects completed the protocol. Compared with placebo, purified anthocyanins moderately reduced HbA1c (−0.14%, 95% CI: −0.23~−0.04%; *p* = 0.005), low-density lipoprotein-c (LDL-c) (−0.2 mmol/L, 95% CI: −0.38~−0.01, *p* = 0.04), apolipoprotein A-1 (apo A1) (0.09 g/L, 95% CI: 0.02~0.17; *p* = 0.02), and apolipoprotein B (apo B) (−0.07 g/L, 95% CI: −0.13~−0.01; *p* = 0.01) according to intention-to-treat analysis. Subgroup analyses suggested that purified anthocyanins were more effective at improving glycemic control, insulin sensitivity, and lipids among patients with elevated metabolic markers. **Conclusions:** The 12-week randomized controlled trials (RCT) in Chinese adults with prediabetes or early untreated diabetes indicated that purified anthocyanins favorably affected glycemic control and lipid profile. Future studies of a longer duration that explore the dose-response relationship among patients with cardiometabolic disorders are needed to confirm our findings.

## 1. Introduction

Diabetes has become a rising challenge in global public health. There is a large burden of diabetes in China, where 10.9% of adults have diabetes and 35.7% have prediabetes [1]. Diabetes management is very cost-effective when initiated at an early stage [2]. Dietary and lifestyle modifications are recommended as the primary strategies for type 2 diabetes prevention [3,4]. The inclusion of dietary phytochemicals is considered a feasible and practical strategy.

Anthocyanins are a subgroup of flavonoids that are responsible for the red-orange to blue-violet pigments in plants (fruits, vegetables, flowers, and grains). Several large scale longitudinal studies have indicated that habitual consumption of anthocyanin-rich foods was associated with reduced risk of type 2 diabetes and cardiovascular diseases [5,6]. A recent meta-analysis [7] of cohort studies reported that the risk of diabetes decreased by 5% with each 7.5 mg/day increment of anthocyanins intake. Evidence from animal experiments demonstrated the beneficial effects of anthocyanins on glycemic control, lipid profile, insulin resistance, oxidative stress, and inflammation [8,9,10]. Results from our in vitro studies further support the biological plausibility that anthocyanins may decrease hyperglycemia and improve lipid profile through activation of adenosine monophosphate (AMP)-activated protein kinase (AMPK) [11] or by regulating transcriptional factor Forkhead box O1 (FoxO1) [12], both of which are molecular mechanisms underlying the critical signaling pathways of glycolipid metabolism.

Several small-scale randomized controlled trials (RCTs) have tested the effects of purified anthocyanins or anthocyanin-rich extracts on glucose homeostasis and insulin sensitivity, but they reported inconsistent findings. Some studies have shown attenuated hyperglycemia [13] and/or hyperlipidemia [14,15], while another showed ineffective results [16]. Most trials on purified anthocyanins were conducted among diseased patients (type 2 diabetes, hypercholesterolemia, nonalcoholic fatty liver disease (NAFLD), etc.) and no RCT has been specifically conducted among individuals with prediabetes. Our previous trials among patients with type 2 diabetes [15] and NAFLD [17] indicated that purified anthocyanins could improve insulin resistance, dyslipidemia, and antioxidant capacity. However, the favorable effects of anthocyanins on cardiometabolic parameters could not eliminate the potential interactions between anthocyanins and medications. Whether these effects could be extended to prediabetic individuals is unclear. Thus, we proposed a randomized controlled trial (RCT) to examine the effects of purified anthocyanins on the metabolic profiles among prediabetes or early untreated diabetes, and sought to determine whether this intervention has important implications for the primary prevention of diabetes and cardiovascular disease.

## 2. Research Design and Methods

### 2.1. Subject Recruitment, Inclusion, and Exclusion Criteria

Participants were recruited from local communities in Guangzhou, China through advertising flyers, medical record review, or clinicians’ recommendations at outpatient clinics. Potential participants were interviewed by trained research staff over the telephone or in person with a structured screening questionnaire. Participants with a recent medical record of hyperglycemia were further invited to attend a clinical visit to receive a standard 3-h 75 g oral glucose tolerance test (OGTT) for confirmation of their eligibility. The enrollment and intervention were conducted at a local hospital from February to October in 2016. The Ethics Committee of Sun Yat-sen University approved the study protocol. All participants signed written informed consent prior to enrollment. The trial was registered at ClinicalTrials.gov (NCT02689765).

Participants were Chinese men and women aged 40–75 years with prediabetes or early untreated diabetes. Prediabetes comprised either impaired fasting glucose (IFG, 5.6–6.9 mmol/L), impaired glucose tolerance (IGT, 2-h post-load glucose of 7.8–11.0 mmol/L), or glycated hemoglobin A1c (HbA1c) of 5.7–6.4% (or 39–46 mmol/mol) according to the American Diabetes Association (ADA) diagnostic criteria [18]. Patients with newly diagnosed type 2 diabetes who exceeded the upper limit of the range (fasting glucose > 6.9 mmol/L, 2-h glucose > 11.0 mmol/L, or HbA1c > 6.4%) were also recruited if hypoglycemic therapy was not required by a clinician’s suggestion.

Participants were excluded if they had a medical history of diabetes, untreated thyroid disease, polycystic ovarian syndrome, serious liver or kidney dysfunction, were currently suffering from acute or chronic infectious diseases or traumatic injury or surgeries, or were currently taking or in the preceding six months had taken hypoglycemic or weight reduction agents, were using glucocorticoids, were lactating or pregnant women, or had a known allergy to anthocyanins or berries.

### 2.2. Study Design, Intervention, Randomization, and Blinding

This was a 12-week CONSORT (Consolidated Standards of Reporting Trials) compliant, randomized, double-blind, placebo-controlled trial. A total of 160 eligible participants with either prediabetes or early untreated diabetes were recruited and randomly assigned to two intervention arms to receive either purified anthocyanins (320 mg/day) or placebo. The dosage was determined based on our previous clinical trials in patients with type 2 diabetes [15] and NAFLD [17] that reported improvement on glycemic control and posed no risk of adverse effects.

A list of random numbers was generated by a computer program. Research staff were not involved in the randomization or the labeling work. The serial numbers and the corresponding supplements were assigned to the eligible subjects in the order of final enrollment into the trial. Participants, investigators, and laboratory technicians were blinded to the treatment assignments until the conclusion of the trial.

### 2.3. Supplements Preparation

Anthocyanins (Medox) and placebo capsules were provided by the Biolink Group. Each capsule of Medox contains 80 mg anthocyanins, which comprises 17 different natural anthocyanins purified from bilberry (*Vaccinium myrtillus*) and blackcurrant (*Ribes nigrum*), refer to Supplemental Tables S1–S3 for the ingredients of anthocyanins capsules. Anthocyanin capsules also contain 4% pullulan, maltodextrin, and citric acid to maintain the stability of anthocyanins, while the placebo capsules contain only pullulan and maltodextrin [19]. The anthocyanin and placebo capsules had identical weight, appearance, and package. Participants were instructed to consume two capsules twice daily preferably 30-min after breakfast and supper. They were asked to maintain their usual dietary intakes and physical activities. They were required not to use any supplements containing anthocyanins and to avoid consumption of anthocyanin-rich foods (red wine, grapes, blueberry, etc.) during the intervention period. Supplements were delivered to subjects every two weeks after randomization.

### 2.4. Data Collection

Individual information was collected by trained research staff via face-to-face interview based on a structured questionnaire on socio-demographic data, medical history, uses of medications, dietary habits, and physical activities. Overnight fasting (8–10 h) venous blood samples were collected between 8:00 and 9:00 a.m., and then a standard 3-h OGTT was performed at both baseline and the end of the treatment. Blood samples were collected at 0, 30, 60, 120, and 180 min after 75 g oral glucose challenge, and were centrifuged at 3000× *g* 4 °C for 15-min to isolate serum within 2-h. Each participant’s serum sample was divided into several aliquots and stored at −85 °C until analysis. Baseline and follow-up specimens from the same individuals were measured in one batch to minimize inter-assay variability.

### 2.5. Primary and Secondary Outcome Measures

The primary outcomes were glycated hemoglobin A1c (HbA1c) and the kinetic parameters of glucose, insulin, and C-peptide by 3-h OGTT. The secondary outcome was a lipid profile including total cholesterol (TC), triglyceride (TG), high-density lipoprotein (HDL) and low-density lipoprotein (LDL) cholesterol, apolipoprotein A-1 (apo A1), and apolipoprotein B (apo B). The areas under the curves (AUC) for glucose, insulin, and C-peptide were calculated according to the trapezoidal rule. The index of hepatic insulin clearance was calculated by the AUC of the C-peptide to insulin ratio [20]. The insulin secretion function of β-cells was estimated by the insulinogenic index (∆_Insulin30min_/∆_Glucose30min_) [21]. The homoeostasis model assessment of insulin resistance (HOMA-IR) and β-cell function (HOMA-β) were calculated based on fasting glucose (FG) and fasting insulin (FIns) [21]: HOMA-IR = FIns (mU/mL) × FG (mmol/L)/22.5; HOMA-β = FIns × 20/(FG-3.5). The exploring outcomes were inflammatory markers or cytokines including uric acid and C-reactive protein (CRP). Biomarkers of liver and kidney function were measured for safety assessment, including serum alanine transaminase (ALT), aspartate aminotransferase (AST), and creatinine. All of the above biomarkers were measured at both baseline and the end of the 12-week treatment.

### 2.6. Biochemical Measurement

HbA1c was measured by cation exchange high pressure liquid chromatography (HPLC, Bio-Rad Laboratories, Hercules, CA, USA). Serum glucose was measured by glucose oxidase method. Serum TC and TG were measured by standardized enzymatic colorimetric methods. Serum HDL-c and LDL-c were measured by enzymatic clearance assay. Serum apo A1, apo B and CRP were measured by immune-turbidimetric method (QuikRead CRP, Orion Diagnostica Oy, Espoo, Finland). Serum glucose and lipids were determined by auto-analyzer (Mindary Automatic Analyzer BS-220, Golden Harvest Industries, Chennai, India) in a certified clinical lab. Serum insulin and C-peptide were determined by chemiluminescent micro-particle immunoassay (Roche Diagnostics, Indianapolis, IN, USA), with sensitivity of 0.2 μU/mL and 0.01 ng/mL, respectively. The coefficients of variation (CVs) for the intra- and inter- assay were 1.33 ± 0.62% and 2.66 ± 1.44% for insulin and 0.85 ± 0.02% and 5.35 ± 0.31% for C-peptide, respectively. For serum glucose and lipids, all the intra- and inter-CVs were less than 5%.

### 2.7. Dietary Intake, Physical Activity, and Anthropometric Assessments

Assessments of dietary intake were based on 3-day food records that were completed by subjects and checked by investigators at baseline and at the end of the trial. Dietary intakes of nutrients were calculated according to the Chinese Food Composition Table [22]. Habitual physical activities were asked about before and after the intervention for the frequency and intensity. Body weight, height, waist, and hip circumference, and blood pressure were measured according to the standard protocols.

### 2.8. Compliance Assessment

Participants were asked to return their rest capsules every two weeks for general visits and to receive new capsules for the next interval. At each regular visit, patients were specifically asked about adverse events. Adverse event was defined as any discomfort during the intervention. Compliance was assessed by counting the unused capsules at each visit. Good compliance was defined as consuming more than 80% of the provided capsules and completing all assessments and sample collections.

### 2.9. Sample Size Planning

Sample size estimation was based on our previous clinical trial using purified anthocyanins as a treatment [15], in which the changes in standard deviation (SD) for HbA1c and fasting glucose were 0.2% (SD 0.4%) and 0.4 mmol/L (SD 0.6 mmol/L), respectively. A sample size of 78 per group would provide 80% power to detect a significant change in HbA1c by 0.2% (2.2 mmol/mol or 3% percent change) or fasting glucose by 0.4 mmol/L (5% percent change), using a conventional assumption of a two-tailed α level of 0.05. Allowing for a 20% drop-out rate, we planned to recruit 80 subjects per group.

### 2.10. Statistical Analysis

Statistical analysis was performed using SPSS 20 (SPSS Inc., Chicago, IL, USA). Baseline characteristics were compared between the two intervention groups to determine their baseline comparability. Data were analyzed according to an intention-to-treat (ITT) principle, which included all 160 randomized subjects. Secondary analyses comprised a per-protocol (PP) analysis which included 138 subjects of good compliance, with the exclusion of withdrawal (*n* = 20) and poor compliance (*n* = 2). Last observation carried forward (LOCF) was used for processing the missing values in the ITT analysis, and the PP analysis did not use any imputation method. The net changes at 12-weeks in outcome variables between the two study groups were compared by both *t*-test and analysis of covariance (ANCOVA) with adjustment of baseline parameters (baseline value, age, sex, and medications for lowering blood pressure and lipids). The Benjamini-Hochberg (B-H) procedure of false discovery rate (FDR) was adopted for multiple testing corrections with an FDR of 0.2.

Stratification analyses were conducted to test whether the effect of anthocyanins differed across various subgroups including high and low levels of fasting glucose (<5.6 vs. ≥5.6 mmol/L), HOMA-IR (<2.9 vs. ≥2.9), HOMA-β (<89.0 vs. ≥89.0), TG (<1.7 vs. ≥1.7 mmol/L), LDL-c (<3.12 vs. ≥3.12 mmol/L), and gender (men vs. women). Before the subgroup analyses, we tested the effect modification by adding an interaction term of treatment and subgroup variables to the univariate models.

## 3. Results

### 3.1. Subject Characteristics at Baseline and Follow-Up

**Figure 1:**
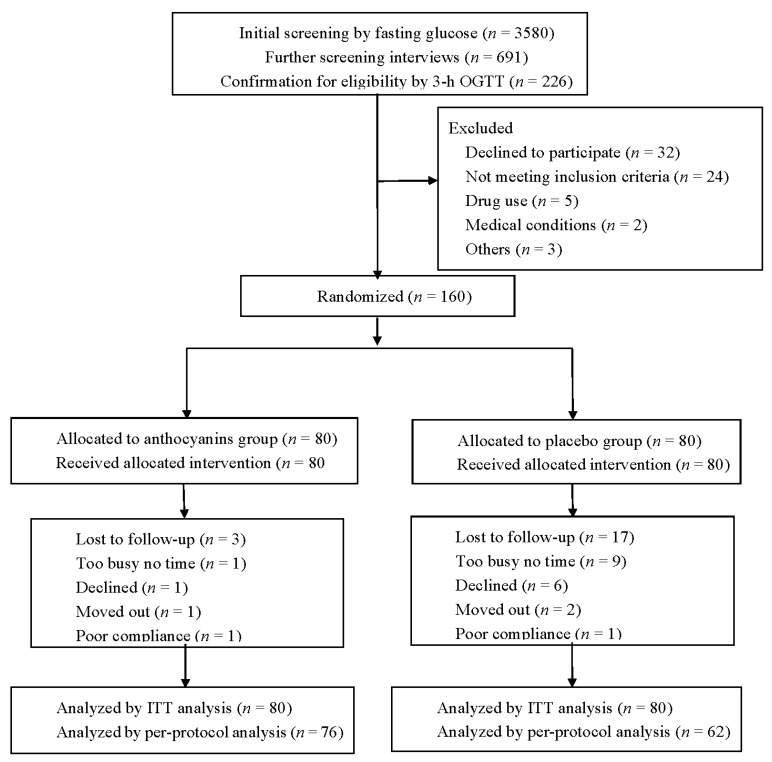
The CONSORT flowchart of the study. ITT = intention-to-treat.

A total of 160 participants were randomly allocated into two study arms. Twenty participants (12.5%) withdrew from the study during the follow-up, and two were in poor compliance. More dropouts were observed in the placebo group (*n* = 13) than the anthocyanins group (*n* = 7), but no significant difference was observed in the proportion of valid completers among the two groups (*p* = 0.160). Detailed reasons for withdrawal are indicated in Figure 1. A total of 140 subjects attended the final visit and 138 received the final 3-h OGTT test.

Baseline measurements were performed before the randomization. Participants in the two groups were comparable in terms of age, gender, education, medical history, body weight, body mass index (BMI), waist to hip ratio (WHR), systolic and diastolic blood pressure (BP), smoking, habitual alcohol drinking, sports activity, and dietary intake of total energy and nutrients (Table 1). No significant difference was observed between the two groups in anthropometric markers and dietary intake of nutrients and anthocyanins in either the baseline or the follow-up data (Tables S4 and S5).

### 3.2. Effects of Purified Anthocyanins on Markers of Glycemic Control and Insulin Resistance

The baseline parameters for glycemic control, insulin resistance, and lipids were comparable between the two study groups. Significant differences were observed in the net changes of HbA1c (−0.14%, 95% CI: −0.23~−0.04%; *p* = 0.005), LDL-c (−0.2 mmol/L, 95% CI: −0.38~−0.01, *p* = 0.04), apo A1 (0.09 g/L, 95% CI: 0.02~0.17; *p* = 0.02), and apo B (−0.07 g/L, 95% CI: −0.13~−0.01; *p* = 0.01), but significant differences were not seen in other biomarkers (Table 2 and Table 3). A similar pattern was observed among 138 valid completers (data will be submitted on request). There was a significant reduction in insulin resistance within the anthocyanins group, but a null change in the placebo group, suggesting the potential ability of these extracts to improve insulin secretion and sensitivity.

### 3.3. Subgroup Analysis

Since no significant interaction was observed between gender and treatment, the results were reported for both men and women. Stratified analyses (Tables S6–S10) showed that purified anthocyanins were more effective on improving glycemic control, insulin sensitivity, and lipid profiles among patients with elevated markers. Compared with placebo, HbA1c, serum fasting glucose, 2 h post-load C-peptide, LDL-c, and apo B were significantly decreased in subjects with FG ≥ 5.6 mmol/L (*P_interaction_* < 0.001); HbA1c, fasting, and post-load C-peptide, AUC of glucose, insulin, and C-peptide were notably reduced in subjects with TG ≥ 1.7 mmol/L (*P_interaction_* < 0.001); HbA1c, fasting glucose and apoA1 levels were notably improved in subjects with HOMA-β < 89.04 (*P_interaction_* < 0.001). The decrease in HbA1c, LDL-c, and apo B were more remarkable in subjects with HOMA-IR ≥ 2.89 (*P_interaction_* < 0.001).

### 3.4. Adverse Events

We documented ten adverse events reported by the participants, three in the placebo and seven in the anthocyanins group. The major adverse events were: dark stool (*n* = 5), insomnia (*n* = 1), abdominal pain (*n* = 1), diarrhea (*n* = 1), dizziness (*n* = 1), and skin rash (*n* = 1). Four participants (three in placebo and one in anthocyanins group) withdrew from the trial due to adverse events.

### 3.5. Compliance

By counting the remaining capsules at every visit, the compliance of participants was assessed in both intervention groups. The actual capsule consumption rates (accounting for total provided capsules) were 86.5% in the placebo group and 90.2% in the anthocyanins group.

## 4. Discussion

### 4.1. Summary of the Main Findings

To our knowledge, this is the first RCT specifically conducted among Chinese midlife men and women with prediabetes or early untreated diabetes to investigate the effects of purified anthocyanins on glucose homeostasis, whole-body insulin sensitivity, and other cardiovascular disease (CVD) risk factors. The results indicated that 12-week supplementation of anthocyanins had favorable effects on reductions in HbA1c, LDL-c, and apo B and increases in apo A1. Subgroup analysis further suggested that patients with elevated metabolic markers may obtain more beneficial improvements in cardiometabolic profiles.

### 4.2. Strengths and Implications

This trial has some advantages that may strengthen the study validity. The design was a randomized, placebo-controlled trial and was able to provide the first level of scientific evidence. We strictly followed the principle of random allocation and allocation concealment. The well-balanced baseline characteristics demonstrated successful randomization. Furthermore, the study was conducted among participants with untreated prediabetes or early diabetes which would eliminate the possible masking effect or interactions of anthocyanins and medications. We comprehensively assessed the effect of anthocyanins on whole-body glucose homeostasis and insulin resistance by using a standard 3-h OGTT. Furthermore, participants maintained their usual dietary habits and physical activity patterns throughout the follow-up, which minimized the possible bias from lifestyle modifications and suggested an independent effect of anthocyanins on metabolic profiles.

Current findings were supported by our recent meta-analysis (article in press by *Advance in Nutrition*) on the RCTs of anthocyanins and cardiometabolic health, which reported a favorable change in HbA1c, fasting glucose, LDL-c, and TC, but null effects on fasting insulin, HOMA-IR, and inflammatory markers. However, in most of the included trials, glucose or insulin metabolism were not the primary outcomes and limited studies used purified anthocyanins as a treatment. Similar to our findings, subgroup analyses suggested a more favorable pooled effect on fasting glucose and LDL-c in patients with hyperglycemia or hyperlipidemia. In addition, a more notable effect was observed on HbA1c with a higher dosage (>400 mg/day) of anthocyanins.

### 4.3. Results Explanation

In the present trial, we observed a modest but significant reduction in HbA1c by anthocyanin treatment, but null effects on glucose and insulin. HbA1c reflects the average glucose level during the past two to three months, and has the advantage of much lower variability compared to glucose and insulin allowing small treatment differences to be detected [23]. Evidence has shown that HbA1c measurements alone are sufficient to provide an accurate estimate of fasting glucose as early as within four weeks of starting anti-diabetic therapy [23]. The modest change in HbA1c could be because our trial started with a relatively low level of HbA1c (average 6.0% at baseline), leaving limited room for improvement. Subgroup analysis also indicated higher baseline fasting glucose and lipid levels, as well as a better response to the anthocyanins treatment. This could explain the significant effect of anthocyanins on fasting glucose among diabetic patients but the non-significant effect in our participants. The data support that patients at greatest risk benefit the most [24].

Decreased HbA1c levels have been shown to reduce microvascular and macrovascular complications after long-term follow-up [25]. According to a 15-year cohort study in the general population of Japan [26], the observed HbA1c reduction in our trial (−0.14% of absolute change or −2.3% of relative change in HbA1c by ITT results) could translate into a 10.1% reduction in all-cause mortality, a 24.5% reduction in CVD death, and 30.7% and 32.2% reductions in the risk of coronary heart disease and cerebral infarction, respectively. Thus, even the modest change in HbA1c as observed in our trial still has important public health implications.

The modest net reduction in HbA1c observed in our study is similar to that achieved by other dietary or lifestyle interventions: −0.18% by a high-cereal fiber diet for six months in type 2 diabetes patients [27], −0.30% by maqui berry extracts for three months in subjects with prediabetes [28], and −0.18% by the PRECEDE (Predisposing, Reinforcing, Enabling, Causes in Educational Diagnosis, and Evaluation) model of health education for two years in type 2 diabetes patients [29]. In addition, reliance on HbA1c remains a primary efficacy endpoint with even a modest change of 0.3~0.4% for diabetes drug development by the United States Food and Drug Administration (FDA) [30].

Similar to our findings, several RCTs [28,31,32] using anthocyanin extracts or other polyphenols also reported a significant reduction in HbA1c, but not in fasting glucose and insulin resistance. The reduction in HbA1c was not accompanied by changes in insulin sensitivity, which could imply that the effects on serum glucose may be through insulin-independent pathways. This is consistent with the results from a study evaluating the effects of fermented blueberry juice on glucose uptake and the AMPK pathway, the latter of which regulates glucose transport in murine muscle cells [33]. AMPK could inhibit 3-hydroxy-3-methylglutaryl-coenzyme A (HMG-CoA) reductase [34], the limiting enzyme of cholesterol synthesis. Therefore, the activation of AMPK would lead to lower cholesterol levels. In our previous experimental study, anthocyanins also inhibited adipocyte lipolysis by blocking FoxO1-mediated transcription of adipose triglyceride lipase (ATGL) [35], which is the major lipase involved in TG breakdown in adipocytes. The above mechanisms may explain the combined changes in glycolipid metabolism biomarkers.

Although statistical significance was not reached with ITT analysis for markers of insulin resistance, exploration of subgroup analysis among patients with elevated cardiometabolic markers suggested a significant favorable effect of purified anthocyanins on fasting glucose and insulin regulation. The findings are also supported by our previous RCT among diabetic patients [15] or patients with NAFLD [17] using the same anthocyanin profile and dosage. The current trial extends the findings to a prediabetic population.

The favorable improvement in lipids (LDL-c, apo A1, and apo B) could have implications for lowering cardiovascular risk in patients with pre- or early diabetes because the plasma concentrations of atherogenic lipoprotein particles, measured by apo B and apo A1, strongly predict factors related to the development of coronary heart disease compared to non-HDL-c [36]. Apo B, in particular, is a sensitive indicator to alterations in glycemic control [36]. Most RCTs using anthocyanins as a treatment have not reported the effects on apolipoproteins. The reduction in apo B and the increase in apo A1 observed in the present study are a further confirmation of the potential CVD benefits. Several RCTs [37,38,39,40] of lifestyle modifications or the use of other phytochemicals also reported significant improvements in apolipoproteins, but not on other lipids. No significant difference in CRP or other lipids was seen in the present study, possibly due to the relative normality of the baseline lipid levels.

### 4.4. Limitations

This study has several limitations. First, the relatively wide variation in initial fasting glucose and insulin levels relative to HbA1c may reduce the power to detect significant differences in glucose and insulin between groups. We only found favorable changes in HbA1c, LDL-c, and apo A1 and B, but not in other markers. The findings herein are explained with caution since we showed several significant findings among a number of comparisons. However, FDR was applied to adjust for multiple hypotheses and indicated similar results.

Second, like most other RCTs using anthocyanins as treatment, we did not detect anthocyanins or their metabolites in serum samples as a measure of compliance, mainly due to the short half-life of anthocyanins (4 h). Thus, an 8- to 10-h fasting state before blood collection resulted in complete clearance and excretion of anthocyanins [41].

Another limitation of this work was the possible incomplete blinding in the placebo group, which may result in more drop-outs. However, LOCF provides conservative results on active treatment if participants in the placebo group drop out early [42]. Thus, the early dropout in the placebo group seems unlikely to affect the findings.

Finally, the dose-response relationship was not tested in our study. In addition, the relatively short duration of our trial may be the reason for the non-significant findings in insulin resistance. However, the meta-analysis [7] indicated that trials using anthocyanin extracts or anthocyanin-rich foods of a shorter duration than ours had a beneficial effect on insulin regulation. Future longer duration studies are warranted since an increased length of treatment may achieve statistical and clinical relevance, especially for the modest efficacy of treatment achieved with phytochemicals.

In conclusion, the 12-week RCT in Chinese adults with prediabetes or early untreated diabetes indicated that purified anthocyanins have beneficial effects on glycemic control and lipid regulation. Future studies of a longer duration that explore the dose-response relationship among patients with cardiometabolic disorders are warranted to confirm our findings.

## Figures and Tables

**Table 1:** Baseline characteristics of the 180 participants in anthocyanins and placebo groups.

	Anthocyanins (*n* = 80)	Placebo (*n* = 80)	*p*
**Demographics**			
Age (year)	60.8 ± 7.9	61.2 ± 6.9	0.724
Gender (male/female)	25/55	29/51	0.504
Education attainment			0.676
Primary school	5 (6.3%)	8 (10%)	
Middle school	51 (63.7%)	48 (60%)	
College	24 (30%)	24 (30%)	
Occupations			0.207
Professionals/technicians	41 (51.3%)	43 (53.8%)	
Sales/workers/farmers	33 (41.2%)	25 (31.2%)	
Others	6 (7.5%)	12 (15%)	
**Dietary intake**			
Total energy (kcal/day)	1753.39 ± 693.75	1819.26 ± 687.87	0.547
Grains and cereals (g/day)	373.4 ± 209.9	395.2 ± 157.9	0.460
Vegetables (g/day)	320.46 ± 202.57	331.51 ± 190.75	0.723
Fruits (g/day)	85.82 ± 176.4	80.79 ± 114.24	0.831
Anthocyanins (mg/day)	10.0 ± 6.6	10.3 ± 4.7	0.791
**Other lifestyle factors**			
Current smoking (%)	12 (15%)	8 (10%)	0.339
Regular alcohol drinking (%)	4 (5%)	4 (5%)	1.000
Sports			0.516
1~3 times/week	29 (36.3%)	33 (42.3%)	
4~7 times/week	51 (63.7%)	47 (58.7%)	
**Anthropometrics**			
Weight (Kg)	63.7 ± 11.9	63.1 ± 10.5	0.742
BMI (Kg/m^2^)	24.7 ± 3.2	24.8 ± 3.4	0.762
WC (cm)	87.8 ± 9.3	88.0 ± 8.9	0.880
WHR	0.905 ± 0.054	0.905 ± 0.055	0.981
**Blood pressures (BP, mmHg)**			
Systolic BP	133.1 ± 16.5	133.0 ± 21.2	0.977
Diastolic BP	78. 8 ± 9. 9	78.7 ± 9.5	0.950

Results are presented as mean ± standard deviation for continuous variables and *n* (%) for categorical variables. The anthocyanins only represent the subjects’ daily dietary intakes, not the dose of supplemental capsules provided. BMI: body mass index; WC: waist circumference; WHR: waist to hip ratio; BP: blood pressure.

**Table 2:** Changes in markers of glycemic control and lipid profile after 12-week treatment by intention-to-treat analysis.

	Anthocyanins (*n* = 80)			Placebo (*n* = 80)			Net Changes (95% CI)	*P_t-test_*	*P_ANCOVA_*
	Baseline	12 Weeks	Change	Baseline	12 Weeks	Change			
**Markers on glycemic control**									
HbA1c (%)	6.13 ± 0.59	5.84 ± 0.49	−0.29 ± 0.35 *	5.96 ± 0.61	5.80 ± 0.61	−0.16 ± 0.24 *	−0.14 (−0.23 ± −0.04) ^†,‡^	0.005	0.024
Fasting glucose (mmol/L)	6.15 ± 0.87	6.19 ± 0.68	0.04 ± 0.95	6.11 ± 0.61	6.26 ± 0.71	0.15 ± 0.60 *	−0.11 (−0.35 ± 0.14)	0.405	0.444
2-h glucose (mmol/L)	9.82 ± 3.33	10.55 ± 3.00	0.72 ± 2.69 *	8.90 ± 3.38	9.46 ± 3.25	0.57 ± 2.22 *	0.16 (−0.61 ± 0.93)	0.686	0.112
Fasting insulin (μU/mL)	11.55 ± 6.23	11.14 ± 6.47	−0.41 ± 5.09	11.99 ± 5.90	11.89 ± 6.90	−0.10 ± 3.65	−0.31 (−1.69 ± 1.08)	0.660	0.600
Fasting C-Peptide (ng/mL)	2.43 ± 0.98	2.46 ± 0.90	0.03 ± 0.70	2.49 ± 1.01	2.54 ± 0.95	0.05 ± 0.58	−0.01 (−0.21 ± 0.19)	0.913	0.764
**Lipid profile**									
TC (mmol/L)	6.11 ± 1.19	5.99 ± 1.24	−0.12 ± 1.13	6.02 ± 1.20	6.04 ± 1.26	0.03 ± 0.89	−0.15 (−0.47 ± 0.17)	0.358	0.331
TG (mmol/L)	1.72 ± 1.02	1.81 ± 1.26	0.10 ± 1.03	1.73 ± 1.24	1.87 ± 1.55	0.14 ± 1.26	−0.04 (−0.4 ± 0.32)	0.820	0.676
HDL-c (mmol/L)	1.47 ± 0.38	1.29 ± 0.40	−0.17 ± 0.31 *	1.46 ± 0.34	1.30 ± 0.32	−0.16 ± 0.21 *	−0.02 (−0.1 ± 0.07)	0.694	0.731
LDL-c (mmol/L)	3.39 ± 0.91	3.10 ± 0.69	−0.29 ± 0.66 *	3.30 ± 0.88	3.20 ± 0.79	−0.09 ± 0.53	−0.2 (−0.38 ± −0.01) ^†,‡^	0.040	0.040
Apo A1 (g/L)	1.59 ± 0.32	1.60 ± 0.32	0.01 ± 0.28	1.65 ± 0.30	1.56 ± 0.23	−0.08 ± 0.23 *	0.09 (0.02 ± 0.17) ^†^	0.020	0.058
Apo B (g/L)	1.16 ± 0.24	1.08 ± 0.21	−0.08 ± 0.18 *	1.14 ± 0.25	1.13 ± 0.28	−0.01 ± 0.18	−0.07 (−0.13 ± −0.01) ^†,‡^	0.015	0.010
LDL-c/HDL-c	1.33 ± 1.07	1.71 ± 1.57	0.18 ± 0.67 *	1.34 ± 1.24	1.70 ± 1.96	0.24 ± 0.53 *	−0.06 (−0.25 ± 0.13)	0.537	0.491

Data are presented as mean ± standard deviation. * *p* < 0.05 by paired *t*-test with comparison of the difference between baseline and 12 week data. ^†^ *p* < 0.05 by independent samples *t*-test with comparison of the difference of net changes between the two groups. ^‡^ *p* < 0.05 by ANCOVA adjusting for covariates (baseline value, age, sex, and medications for lowering blood pressure and lipids) with comparison of the net changes between the two groups. HbA1c: glycated hemoglobin A1c; TC: Total cholesterol; TG, Triglycerides; HDL-c: high density lipoprotein cholesterol; LDL-c: low-density lipoprotein cholesterol; Apo A1: apolipoprotein A-1; Apo B: apolipoprotein B.

**Table 3:** Changes in insulin sensitivity, inflammatory, and safety markers after 12-week treatment by intention-to-treat analysis.

	Anthocyanins (*n* = 80)			Placebo (*n* = 80)			Net Change (95% CI)	*P_t-test_*	*P_ANCOVA_*
	Baseline	12 Weeks	Change	Baseline	12 Weeks	Change			
**Markers of Insulin sensitivity**									
HOMA-IR	3.18 ± 1.95	3.12 ± 1.94	−0.06 ± 1.76	3.30 ± 1.75	3.35 ± 2.09	0.05 ± 1.23	−0.11 (−0.59 ± 0.36)	0.636	0.568
HOMA-β	94.87 ± 56.35	84.45 ± 45.64	−10.41 ± 44.86 *	97.54 ± 77.53	89.03 ± 52.53	−8.51 ± 54.22	−1.91 (−17.45 ± 13.63)	0.809	0.563
AUC_Glucose_	28.82 ± 6.42	30.47 ± 5.99	1.65 ± 5.39 *	29.11 ± 6.35	29.11 ± 6.35	1.68 ± 4.69 *	−0.02 (−1.60 ± 1.56)	0.977	0.412
AUC_Insulin_	206.66 ± 99.75	235.41 ± 134.69	28.76 ± 119.08 *	227.34 ± 108.68	255.48 ± 146.38	28.14 ± 87.83 *	0.61 (−32.06 ± 33.29)	0.970	0.971
AUC_C-Peptide_	26.32 ± 6.51	27.05 ± 7.06	0.74 ± 5.60	27.14 ± 6.87	28.30 ± 8.20	1.16 ± 5.15 *	−0.42 (−2.10 ± 1.26)	0.619	0.524
Insulinogenic index	10.94 ± 8.96	11.28 ± 8.99	0.34 ± 6.21	12.90 ± 8.68	13.19 ± 9.18	0.30 ± 5.95	0.04 (−1.86 ± 1.94)	0.964	0.636
Insulin clearance	7.09 ± 2.04	6.47 ± 2.12	−0.61 ± 1.58 *	6.49 ± 1.61	6.15 ± 1.70	−0.34 ± 1.15 *	−0.27 (−0.70 ± 0.16)	0.216	0.625
**Inflammatory markers**									
CRP (mg/L)	1.90 ± 1.97	2.24 ± 2.70	0.34 ± 2.05	2.20 ± 2.87	2.65 ± 3.72	0.45 ± 3.93	−0.11 (−1.09 ± 0.87)	0.821	0.522
Uric Acid (umol/L)	390.4 ± 111.1	365.1 ± 115.8	−25.3 ± 98.3 *	352.3 ± 92.8	337.3 ± 103.0	−15.0 ± 58.9 *	−10.2 (−35.5 ± 15.1)	0.426	0.646
**Markers on liver and renal function**									
ALT (U/L)	21.58 ± 8.66	20.40 ± 9.30	−1.18 ± 7.40	23.63 ± 8.78	21.01 ± 9.20	−2.62 ± 5.11 *	1.45 (−0.54, 3.43)	0.153	0.387
AST (U/L)	22.05 ± 5.68	23.20 ± 6.15	1.15 ± 5.08 *	22.78 ± 5.36	22.71 ± 5.66	−0.07 ± 3.69	1.22 (−0.17, 2.60)	0.085	0.150
Creatinine (umol/L)	74.47 ± 20.40	74.60 ± 20.64	0.13 ± 7.92	71.56 ± 20.21	72.71 ± 22.17	1.15 ± 8.05	−1.02 (−3.51,1.48)	0.421	0.550

Data are presented as mean ± standard deviation. * *p* < 0.05 by paired *t*-test with comparison of the difference between baseline and 12-week data. HOMA-IR: homoeostasis model assessment of insulin resistance; HOMA-IR = FIns(mU/mL) × FG(mmol/L)/22.5. FIns: Fasting insulin; FG: fasting glucose; HOMA-β: homoeostasis model assessment of β-cell function; HOMA-β = FIns × 20/(FG-3.5). AUC, area under the curve by 3-h oral glucose tolerance test, were calculated according the trapezoidal rule. CRP: C-reactive protein. Insulinogenic index = (Insulin30min-Insulin0min)/(Glucose30min-Glucose0min). Insulin clearance = AUC_C-Peptide_/AUC_Insulin_ (Insulin 1 μU/mL * 6.945 = 1 pmol/L, C-Peptide 1 ng/mL * 333.33 = 1 pmol/L). ALT: alanine transaminase; AST: aspartate transaminase.

## Supplementary Materials

The following are available online at www.mdpi.com/2072-6643/9/10/1104/s1, Tables S1–S3: ingredients of anthocyanins capsules, Tables S4–S5: changes in anthropometrics and dietary nutrients, Tables S6–S10: subgroup analysis results.
