# Supplementary material for: Role of Purified Anthocyanins in Improving Cardiometabolic Risk Factors in Chinese Men and Women with Prediabetes or Early Untreated Diabetes—A Randomized Controlled Trial
Source: Nutrients. 2017 Oct 10;9(10):1104. doi: 10.3390/nu9101104 (PMC5691720; doi:10.3390/nu9101104)
Supplement: Supplementary file 1 [file nutrients-09-01104-s001.zip › nutrients-223266-supplementary.pdf]

The Medox and placebo capsules were similar and processed and packaged at the Biolink Group's own processing factory in Sandnes, Norway. Medox is a veg-encapsulated anthocyanin extract from wild Norwegian bilberries (Vaccinium myrtillus) and black currants (Ribes nigrum). The anthocyanin capsules also contained pullulan, maltodextrin, and citric acid (which occupied for 4% per capsule and was helpful to maintain stability of anthocyanins), whereas the placebo capsules only contained pullulan and maltodextrin. The specific ingredients of anthocyanins capsules are shown in Supplemental Table S1.

Supplemental Table S1 The composition of anthocyanins capsules

Components	Content
Bilberry (Vaccinium myrtillus) skin	45-52%
Blackcurrant (Ribes nigrum) skin	12-13%
Pullulan	20-28%
Maltodextrin	11-12%
Citric acid	3-4%

European bilberry and black currant are rich in a variety of anthocyanins, mainly concentrated in the peel. According to literatures, bilberry contains 15 kinds of natural anthocyanins; blackcurrant contains 4 kinds of natural anthocyanins, the composition detailed in Table S2. The total amount of anthocyanins in each capsule is 80mg, which is a mixture of 3-*O*-rutinosides of cyanidin and delphinidin, and 3-*O*-b-galactosides, 3-*O*-b-glucosides, and 3-*O*-b-arabinosides of cyanidin, peonidin, delphinidin, petunidin, and malvidin. The relative contents of various anthocyanins are shown in Table S3.

Supplemental Table S2 The anthocyanins contained in Vaccinium myrtillus and Ribes nigrum

No	Anthocyanins	Abbreviations
Vaccinium myrtillus		
1	Delphinidin 3-O-β-glucosides	Dp-3-G
2	Delphinidin 3-O-β-galactosides	Dp-3-gal
3	Delphinidin 3-O-β-arabinosides	Dp-3-A
4	Cyanidin 3-O-β-glucosides	Cy-3-G

5	Cyanidin 3-O-β-galactosides	Cy-3-gal
6	Cyanidin 3-O-β-arabinosides	Cy-3-A
7	Petunidin 3-O-β-glucosides	Pe-3-G
8	Petunidin 3-O-β-galactosides	Pe-3-gal
9	Petunidin 3-O-β-arabinosides	Pe-3-A
10	Peonidin 3-O-β-glucosides	Pn-3-G
11	Peonidin 3-O-β-galactosides	Pn-3-gal
12	Peonidin 3-O-β-arabinosides	Pn-3-A
13	Malvidin 3-O-β-glucosides	Mv-3-G
14	Malvidin 3-O-β-galactosides	Mv-3-gal
15	Malvidin 3-O-β-arabinosides	Mv-3-A
Ribes nigrum		
1	Delphinidin 3-O-β-glucosides	Dp-3-G
2	Cyanidin 3-O-β-glucosides	Cy-3-G
3	Delphinidin 3-O-rutinoside	Dp-3-R
4	Cyanidin 3-O-rutinoside	Cy-3-R

Supplemental Table S3 The relative contents of anthocyanins in Medox capsules

Anthocyanins	Content（%）
Dp-3-G, Dp-3-gal, Dp-3-A	58.0
Cy-3-G, Cy-3-gal, Cy-3-A	33.0
Mv-3-G, Mv-3-gal, Mv-3-A	3.0
Pe-3-G, Pe-3-gal, Pe-3-A	2.5
Pn-3-G, Pn-3-gal, Pn-3-A	2.5
Dp-3-R, Cy-3-R	1.0
Total	100.0

Supplemental Table S4 Changes in anthropometrics and blood pressure at baseline and after 12-week treatment by ITT analysis

Parameters	Anthocyanins(n=80)		Placebo(n=80)		Net change（CI95%）	$P_{t\text{-}test}$	P_{ANCOVA}
	Baseline	12 weeks	Baseline	12 weeks			
**Anthropometrics**							
Weight(Kg)	63.66±11.87	62.89±11.71*	63.08±10.45	62.64±10.52*	-0.33(-0.69, 0.03)	0.073	0.141
BMI(Kg/m2)	24.66±3.18	24.33±3.12*	24.82±3.44	24.60±3.41*	-0.10(-0.3, 0.09)	0.289	0.387
Waist(cm)	87.8±9.27	86.52±8.58*	88.02±8.94	87.68±9.16	-0.94(-2.2, 0.31)	0.140	0.094
WHR	0.905±0.054	0.896±0.050	0.905±0.055	0.908±0.053	-0.01(-0.02, 0.002)	0.088	0.059
NC	34.99±3.07	35.00±3.28	34.96±2.98	35.08±3.06	-0.12(-0.46, 0.22)	0.503	0.716
**Blood pressure**							
SBP(mmHg)	133.09±16.50	129.93±13.76*	133±21.18	131.48±18.62	-1.63(-5.31, 2.04)	0.381	0.400
DBP(mmHg)	78.78±9.89	76.58±9.00*	78.69±9.46	76.55±10.63*	-0.07(-2.34, 2.2)	0.951	0.933

The values are presented as mean ± standard deviation.

** P<0.05 by paired t-test with comparison of the difference between baseline and 12 weeks data.*

$P_{t\text{-}test}$ *was the comparison of net changes between the two groups, compared by independent samples t-test.*

P_{ANCOVA} *was the comparison by analysis of covariates (ANCOVA) adjusting for baseline values (age, sex, medications for lowering blood pressure and lipids).*

BMI, body mass index, WHR: waist to hip ratio, NC, neck circumference, DBP, diastolic blood pressure, SBP, systolic blood pressure.

Supplemental Table S5 Daily dietary intakes of total energy and nutrients at baseline and 12 weeks after treatment.

Energy and nutrients	Anthocyanins(n=80)		Placebo(n=80)		P_{change}
	Baseline	12 weeks	Baseline	12 weeks	
Total energy(kcal/d)	1753.39±693.75	1763.91±585.49	1819.26±687.87	1788.71±645.02	0.721
Protein(g/d)	107.03±46.29	110.85±44.22	114.86±57.35	113.68±56.06	0.567
Carbohydrates(g/d)	271.44±134.05	278.03±118.2	282.51±105.21	270.12±98.68	0.332
Total fat(g/d)	51.46±28.8	54.15±33.34	53.42±31.95	59.45±39.91	0.579
Cholesterol(mg/d)	265.79±167.56	283.31±177.68	262.08±143.74	297.3±173.79	0.553
Vitamin A (IU/d)	503.86±335.1	495.27±248.11	505.74±257.84	505.33±226.7	0.856
Vitamin E (mg/d)	19.94±26.01	19.57±13.19	19.52±18.07	20.52±17.62	0.712
Vitamin C (mg/d)	60.26±38.98	56.78±33.67	62.1±34.94	58.38±29.96	0.966
Dietary fibers (g/d)	16.11±10.9	15.64±6.1	16.52±8.05	16.3±7.35	0.864
Anthocyanins (mg/d)	10.02±6.63	9.88±3.59	10.27±4.7	9.86±4.09	0.756

Food groups and nutrients were calculated based on 3-day dietary records at baseline and at 12 weeks. P values were the comparisons of net changes between the two groups by independent t-test. The anthocyanins only represent the subjects' daily dietary intakes, not the dose of supplemental capsules provided.

Supplemental Table S6 Changes in cardiometabolic parameters after 12-week treatment stratified by baseline fasting glucose levels (≥5.6 vs. <5.6 mmol/L)

	Fasting glucose≥5.6mmol/L			Fasting glucose<5.6 mmol/L		
	Anthocyanins(n=60)	Placebo(n=66)	$P_{t\text{-}test}$	Anthocyanins(n=20)	Placebo(n=14)	$P_{t\text{-}test}$
HbA1c (%)	-0.30±0.38*	-0.17±0.24	0.018	-0.26±0.24	-0.11±0.27	0.093
Fasting glucose(mmol/L)	-0.23±0.83*	0.10±0.60	0.012	0.86±0.83	0.37±0.60	0.067
2-h glucose(mmol/L)	0.56±2.88	0.73±2.09	0.719	1.20±1.98	-0.19±2.69	0.090
Fasting insulin(μU/ml)	-0.61±5.55	-0.41±3.62	0.806	0.18±3.41	1.32±3.59	0.355
2-h insulin(μU/ml)	8.00±52.46	19.15±41.9	0.188	42.04±74.62*	-10.55±39.65	0.022
Fasting C-Peptide(ng/ml)	-0.09±0.71	0.00±0.59	0.418	0.42±0.50	0.25±0.50	0.354
2-h C-Peptide(ng/ml)	0.27±2.41*	1.16±2.33	0.037	1.35±3.47	-0.64±2.54	0.077
$AUC_{Glucose}$	1.23±5.70	1.76±4.85	0.576	2.91±4.24	1.28±4.02	0.268
$AUC_{Insulin}$	15.99±101.47	28.93±75.35	0.415	67.05±157.89	24.43±135.92	0.419
$AUC_{C\text{-}Peptide}$	0.16±5.03	1.48±4.62	0.127	2.46±6.92	-0.36±7.17	0.258

HOMA-IR	-0.26±1.89	-0.06±1.23	0.471	0.53±1.16	0.56±1.16	0.938
HOMA-β	0.66±36.30	-4.93±24.79	0.311	-43.63±52.22	-25.37±120.09	0.549
TC (mmol/L)	-0.22±1.15	0.08±0.93	0.117	0.16±1.05	-0.23±0.68	0.237
TG (mmol/L)	0.20±1.13	0.02±0.91	0.338	-0.20±0.61	0.69±2.25	0.100
HDL-c (mmol/L)	-0.19±0.32	-0.14±0.21	0.388	-0.14±0.29	-0.22±0.20	0.370
LDL-c (mmol/L)	-0.36±0.69*	-0.07±0.57	0.010	-0.08±0.56	-0.21±0.25	0.408
Apo A-1 (g/L)	-0.01±0.28	-0.08±0.24	0.108	0.06±0.28	-0.10±0.16	0.063
Apo B (g/L)	-0.08±0.19*	0.01±0.18	0.006	-0.06±0.17	-0.09±0.15	0.651

Data are presented as mean ± standard deviation.

**P<0.05 by independent samples t-test with comparison of the difference of net changes between the two groups.*

HOMA-IR, homoeostasis model assessment of insulin resistance; HOMA-β, homoeostasis model assessment of β-cell function; AUC, area under the curve of 3-hour oral glucose tolerance test, were calculated according the trapezoidal rule; TC, Total cholesterol; TG, Triglycerides; HDL-c, high-density lipoprotein cholesterol; LDL-c, low-density lipoprotein cholesterol.

Supplemental Table S7 Changes of cardiometabolic parameters after 12-week treatment stratified by baseline triglycerides (≥1.7 vs. <1.7 mmol/L)

	TG≥1.7 mmol/L			TG<1.7 mmol/L		
	Anthocyanins(n=28)	Placebo(n=29)	$P_{t\text{-}test}$	Anthocyanins(n=52)	Placebo(n=51)	$P_{t\text{-}test}$
HbA1c (%)	-0.38±0.37*	-0.17±0.23	0.010	-0.24±0.33	-0.15±0.26	0.105
Fasting glucose(mmol/L)	0.11±0.92	0.14±0.7	0.888	0±0.98	0.15±0.55	0.352
2-h glucose(mmol/L)	0.4±2.81	1.29±1.83	0.161	0.9±2.63	0.15±2.33	0.132
Fasting insulin(μU/ml)	-1.97±5.7	-0.2±3.44	0.166	0.42±4.57	-0.05±3.8	0.569
2-h insulin(μU/ml)	7.54±67.49	32.52±49.31	0.115	21.34±55.86	3.39±34.91	0.054

Fasting C-Peptide(ng/ml)	-0.3±0.73*	0.08±0.62	0.035	0.22±0.61	0.03±0.57	0.1
2-h C-Peptide(ng/ml)	0.21±2.97*	1.83±2.43	0.028	0.71±2.61	0.28±2.3	0.38
$AUC_{Glucose}$	0.43±5.76	3.09±5.26	0.073	2.31±5.12	0.87±4.18	0.12
$AUC_{Insulin}$	-1.4±105.88*	50.31±84.44	0.046	44.99±123.54	15.54±88.03	0.167
$AUC_{C\text{-}Peptide}$	-0.79±5.03*	2.48±4.6	0.013	1.56±5.77	0.41±5.34	0.297
HOMA-IR	-0.6±1.99	0.03±1.19	0.155	0.22±1.58	0.06±1.27	0.574
HOMA-β	-10.26±43.43	-4.2±27.86	0.531	-10.49±46.03	-10.95±64.76	0.967
TC (mmol/L)	-0.12±1.17	0.08±0.86	0.468	-0.12±1.12	0±0.92	0.553
TG (mmol/L)	-0.09±1.44	-0.11±1.21	0.936	0.2±0.72	0.28±1.28	0.67
HDL-c (mmol/L)	-0.1±0.43	-0.13±0.21	0.754	-0.21±0.22	-0.17±0.22	0.358
LDL-c (mmol/L)	-0.26±0.63	-0.15±0.43	0.458	-0.31±0.69	-0.06±0.58	0.053
Apo A-1 (g/L)	0.08±0.27*	-0.05±0.23	0.06	-0.03±0.28	-0.11±0.23	0.121
Apo B (g/L)	-0.05±0.2	0.01±0.18	0.217	-0.09±0.18	-0.02±0.18	0.037

*Data were presented as mean ± standard deviation. Subgroups were stratified by the dyslipidemia standards in China. *P<0.05 by independent samples t-test with comparison of the difference of net changes between the two groups. HOMA-IR, homoeostasis model assessment of insulin resistance; HOMA-β, homoeostasis model assessment of β-cell function; AUC, area under the curve of 3-hour oral glucose tolerance test, were calculated according the trapezoidal rule; TC, Total cholesterol; TG, Triglycerides; HDL-c, high-density lipoprotein cholesterol; LDL-c, low-density lipoprotein cholesterol.*

Supplemental Table S8 Changes of cardiometabolic parameters after 12-week treatment stratified by baseline LDL-c (≥3.12 vs. < 3.12 mmol/L)

	LDL-c≥3.12mmol/L			LDL-c<3.12mmol/L		
	Anthocyanins(n=41)	Placebo(n=43)	$P_{t\text{-}test}$	Anthocyanins(n=39)	Placebo(n=37)	$P_{t\text{-}test}$
HbA1c (%)	-0.27±0.38*	-0.11±0.23	0.026	-0.31±0.31	-0.20±0.25	0.097

Fasting glucose(mmol/L)	0.04±1.09	0.10±0.63	0.758	0.04±0.79	0.20±0.58	0.329
2-h glucose(mmol/L)	0.49±2.98	0.84±1.95	0.523	0.97±2.35	0.24±2.49	0.194
Fasting insulin(μU/ml)	-0.11±6.08	-0.08±4.35	0.978	-0.73±3.83	-0.14±2.68	0.438
2-h insulin(μU/ml)	22.60±60.96	16.24±45.46	0.588	10.10±59.33	11.29±39.94	0.919
Fasting C-Peptide(ng/ml)	0.05±0.74	0.08±0.61	0.833	0.02±0.65	0.00±0.55	0.919
2-h C-Peptide(ng/ml)	0.67±2.63	0.79±2.35	0.815	0.40±2.86	0.9±2.59	0.425
$AUC_{Glucose}$	1.12±6.04	1.66±4.59	0.645	2.21±4.64	1.69±4.87	0.637
$AUC_{Insulin}$	36.13±139.94	18.67±73.49	0.473	21.00±93.53	39.15±101.96	0.421
$AUC_{C\text{-}Peptide}$	0.82±6.29	0.52±4.17	0.795	0.65±4.86	1.91±6.08	0.320
HOMA-IR	0.04±2.09	0.05±1.43	0.989	-0.18±1.36	0.05±0.96	0.404
HOMA-β	-11.35±55.47	-2.84±32.19	0.396	-9.43±30.71	-15.08±71.83	0.654
TC (mmol/L)	-0.34±1.26	-0.17±0.77	0.455	0.10±0.95	0.25±0.99	0.514
TG (mmol/L)	0.26±1.14	0.09±0.89	0.463	-0.07±0.88	0.19±1.60	0.373
HDL-c (mmol/L)	-0.21±0.37	-0.16±0.18	0.467	-0.14±0.23	-0.15±0.25	0.796
LDL-c (mmol/L)	-0.61±0.65*	-0.27±0.46	0.007	0.04±0.50	0.11±0.54	0.593
Apo A-1 (g/L)	0.00±0.31	-0.10±0.22	0.093	0.02±0.25	-0.07±0.25	0.117
Apo B (g/L)	-0.14±0.19*	-0.01±0.17	0.001	-0.02±0.16	-0.01±0.20	0.792

*Data were presented as mean ± standard deviation. Subgroups were stratified by the dyslipidemia standards in China. *$P<0.05$ by independent samples t-test with comparison of the difference of net changes between the two groups. HOMA-IR, homoeostasis model assessment of insulin resistance; HOMA-β, homoeostasis model assessment of β-cell function; AUC, area under the curve of 3-hour oral glucose tolerance test, were calculated according the trapezoidal rule; TC, Total cholesterol; TG, Triglycerides; HDL-c, high-density lipoprotein cholesterol; LDL-c, low-density lipoprotein cholesterol.*

Supplemental Table S9　Changes of cardiometabolic parameters after 12-week treatment stratified by baseline HOMA-IR (≥ 2.89 vs. < 2.89)

	HOMA-IR≥2.89			HOMA-IR<2.89		
	Anthocyanins(n=39)	Placebo(n=41)	$P_{t\text{-}test}$	Anthocyanins(n=41)	Placebo(n=39)	$P_{t\text{-}test}$
HbA1c (%)	-0.37±0.37*	-0.17±0.26	0.006	-0.22±0.31	-0.14±0.23	0.225
Fasting glucose(mmol/L)	0.00±0.67	-0.02±0.53	0.917	0.08±1.16	0.32±0.64	0.262
2-h glucose(mmol/L)	0.38±2.78	0.58±1.77	0.703	1.05±2.59	0.55±2.64	0.397
Fasting insulin(μU/ml)	-0.81±6.86	-0.41±4.32	0.758	-0.04±2.49	0.22±2.82	0.665
2-h insulin(μU/ml)	17.08±77.9	23.29±52.37	0.675	15.97±37.06	4.12±26.98	0.108
Fasting C-Peptide(ng/ml)	-0.12±0.86	-0.08±0.67	0.831	0.18±0.46	0.18±0.44	0.993
2-h C-Peptide(ng/ml)	0.48±3.21	1.31±2.62	0.208	0.59±2.21	0.35±2.19	0.633
$AUC_{Glucose}$	0.90±5.80	1.17±4.95	0.824	2.37±4.94	2.21±4.40	0.881
$AUC_{Insulin}$	40.25±155.57	43.94±108	0.902	17.82±69.02	11.53±56.63	0.658
$AUC_{C\text{-}Peptide}$	0.91±6.74	1.69±5.72	0.580	0.58±4.33	0.61±4.49	0.970
HOMA-IR	-0.25±2.40	-0.09±1.41	0.710	0.11±0.79	0.20±1.01	0.690
HOMA-β	-9.81±48.42	-11.87±74.22	0.884	-10.98±41.79	-4.97±16.99	0.399
TC (mmol/L)	0.02±1.06	0.23±0.98	0.362	-0.26±1.19	-0.18±0.74	0.750
TG (mmol/L)	0.24±1.36	0.15±1.02	0.748	-0.04±0.54	0.13±1.48	0.514
HDL-c (mmol/L)	-0.11±0.36	-0.16±0.22	0.461	-0.23±0.25	-0.15±0.21	0.134
LDL-c (mmol/L)	-0.30±0.51*	-0.04±0.58	0.036	-0.28±0.79	-0.15±0.47	0.377
Apo A-1 (g/L)	0.01±0.29	-0.09±0.22	0.091	0.01±0.26	-0.08±0.24	0.118
Apo B (g/L)	-0.07±0.16*	0.02±0.21	0.024	-0.08±0.20	-0.04±0.14	0.277

*Data were presented as mean ± standard deviation. Subgroups were stratified by the median value. *$P<0.05$ by independent samples t-test with comparison of the difference of net changes between the two groups. HOMA-IR, homoeostasis model assessment of insulin resistance; HOMA-β, homoeostasis model assessment of β-cell function; AUC,*

area under the curve of 3-hour oral glucose tolerance test, were calculated according the trapezoidal rule; TC, Total cholesterol; TG, Triglycerides; HDL-c, high-density lipoprotein cholesterol; LDL-c, low-density lipoprotein cholesterol.

Supplemental Table S10 Changes of cardiometabolic parameters after 12-week treatment stratified by baseline HOMA-β (≥ 89.04 vs. < 89.04)

	HOMA-β≥89.04			HOMA-β<89.04		
	Anthocyanins(n=41)	Placebo(n=38)	$P_{t\text{-}test}$	Anthocyanins(n=39)	Placebo(n=41)	$P_{t\text{-}test}$
HbA1c (%)	-0.31±0.33*	-0.12±0.26	0.006	-0.27±0.36	-0.19±0.22	0.234
Fasting glucose(mmol/L)	0.36±0.87*	0.02±0.48	0.033	-0.29±0.93*	0.27±0.68	0.003
2-h glucose(mmol/L)	0.79±2.81	0.82±1.49	0.948	0.66±2.59	0.33±2.74	0.578
Fasting insulin(μU/ml)	-1.05±6.72	0.04±4.29	0.392	0.25±2.34	-0.24±2.97	0.409
2-h insulin(μU/ml)	23.29±79.14	24.51±53.83	0.936	9.38±28.64	3.90±25.55	0.369
Fasting C-Peptide(ng/ml)	0.05±0.90	-0.03±0.69	0.664	0.02±0.40	0.12±0.46	0.312
2-h C-Peptide(ng/ml)	0.87±3.44	1.34±2.89	0.506	0.19±1.68	0.37±1.86	0.647
$AUC_{Glucose}$	1.70±5.95	1.65±4.31	0.968	1.61±4.82	1.70±5.09	0.932
$AUC_{Insulin}$	46.56±155.86	48.49±110.14	0.949	10.04±56.63	8.79±54.07	0.920
$AUC_{C\text{-}Peptide}$	1.54±6.87	1.98±6.07	0.760	-0.10±3.77	0.38±4.01	0.580
HOMA-IR	-0.15±2.35	0.07±1.39	0.611	0.02±0.80	0.03±1.08	0.989
HOMA-β	-27.4±53.79	-12.05±76.13	0.299	7.45±22.36*	-5.14±16.63	0.005
TC (mmol/L)	0.10±1.10	0.04±0.91	0.795	-0.36±1.13	0.01±0.89	0.109
TG (mmol/L)	0.05±1.25	0.36±1.67	0.357	0.15±0.75	-0.07±0.62	0.169
HDL-c (mmol/L)	-0.09±0.37	-0.17±0.21	0.194	-0.27±0.21*	-0.14±0.22	0.011
LDL-c (mmol/L)	-0.22±0.54	-0.12±0.52	0.404	-0.36±0.78	-0.07±0.54	0.051

Apo A-1 (g/L)	0.06±0.28*	-0.08±0.18	0.011	-0.04±0.27	-0.09±0.27	0.424
Apo B (g/L)	-0.07±0.17	-0.02±0.21	0.228	-0.09±0.20*	0.01±0.16	0.029

*Data were presented as mean ± standard deviation. Subgroups were stratified by the median value. *P<0.05 by independent samples t-test with comparison of the difference of net changes between the two groups. HOMA-IR, homoeostasis model assessment of insulin resistance; HOMA-β, homoeostasis model assessment of β-cell function; AUC, area under the curve of 3-hour oral glucose tolerance test, were calculated according the trapezoidal rule; TC, Total cholesterol; TG, Triglycerides; HDL-c, high-density lipoprotein cholesterol; LDL-c, low-density lipoprotein cholesterol.*
